# Ceramide Acyl Chain Length and Its Relevance to Intracellular Lipid Regulation

**DOI:** 10.3390/ijms23179697

**Published:** 2022-08-26

**Authors:** Qing Wei Calvin Ho, Xiaofeng Zheng, Yusuf Ali

**Affiliations:** 1 Lee Kong Chian School of Medicine, Nanyang Technological University Singapore, Singapore 308232, Singapore; 2 Department of Endocrinology and Metabolism, Center for Diabetes and Metabolism Research, West China Hospital, Sichuan University, Chengdu 610041, China; 3 Singapore Eye Research Institute (SERI), Singapore General Hospital, Singapore 168751, Singapore

**Keywords:** ceramides, lipid droplets, intracellular fatty acids, metabolic disease, skin disorders

## Abstract

Ceramides are a class of sphingolipids which are implicated in skin disorders, obesity, and other metabolic diseases. As a class with pleiotropic effects, recent efforts have centred on discerning specific ceramide species and their effects on atopic dermatitis, obesity, type 2 diabetes, and cardiovascular diseases. This delineation has allowed the identification of disease biomarkers, with long acyl chain ceramides such as C16- and C18-ceramides linked to metabolic dysfunction and cardiac function decline, while ultra-long acyl chain ceramides (>25 carbon acyl chain) were reported to be essential for maintaining a functional skin barrier. Given the intricate link between free fatty acids with ceramides, especially the de novo synthetic pathway, intracellular lipid droplet formation is increasingly viewed as an important mechanism for preventing accumulation of toxic ceramide species. Here, we review recent reports of various ceramide species involved in skin abnormalities and metabolic diseases, and we propose that promotion of lipid droplet biogenesis can be seen as a potential protective mechanism against deleterious ceramides.

## 1. Introduction

Recently, there has been considerable interest in a subtype of sphingolipids known as ceramides, and in particular ceramide level derangements in skin and metabolic health. While the pleiotropic effects of ceramides have been reviewed extensively [1,2,3,4,5,6,7,8,9], knowledge of its abundance in relation to intracellular lipid partitioning and regulation remains patchy. This review starts with a summary on the significance of ceramides in well-studied areas such as skin abnormalities and metabolic disease, before a focus on current knowledge surrounding intracellular lipid regulation and its relation to intracellular ceramide levels.

## 2. Ceramides Synthesis

Ceramides are a class of bioactive lipids which comprise of a sphingoid base (commonly sphingosine) and a fatty acyl chain, and they were largely generalised as ER stress, apoptotic triggering molecules in various cell types [10,11,12]. The toxicity of a ceramide species is linked to the length, and the degree of saturation, of fatty acyl chains. For example, saturated C18:0 and C16:0 ceramides were more toxic to HeLa cells compared to C18:1 and C24:1 ceramides [13]. Of note, the sphingoid base of the sphingolipids is also a crucial determinant of its toxicity, as dihydroceramide, which lacks a double bond in its sphingoid base in comparison to ceramides, does not share similar cytotoxicity as observed in ceramides [14,15].

**Figure 1:**
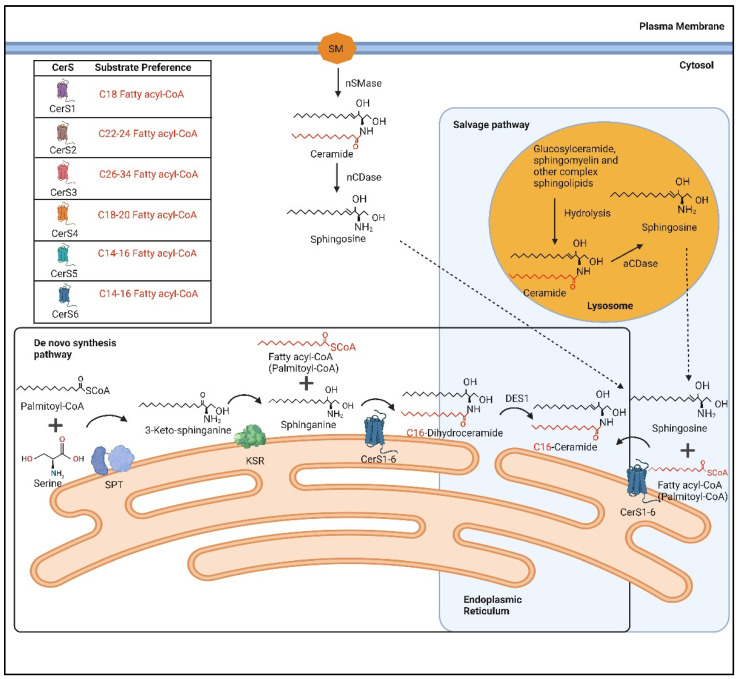
An illustration of the different ceramides generated either through the de novo synthesis, or the salvage pathway. aCDase: acid ceramidase; nCDase: neutral ceramidase; nSMase: neutral sphingomyelinase; CerS: ceramide synthases; DES1: dihydroceramide desaturase-1; KSR: 3-ketosphinganine reductase; SPT: serine palmitoyltransferase. Created with BioRender.com.

The generation of ceramides occurs through three pathways: the de novo synthesis pathway; the salvage pathway; and the sphingomyelinase pathway. The de novo synthesis pathway occurs at the ER and begins with the condensation of serine with palmitoyl-CoA to form 3-keto-sphinganine, a process catalysed by serine-palmitoyltransferase (SPT) (Figure 1) [16]. Subsequently, 3-keto-sphinganine is reduced to sphinganine by 3-keto-sphinganine reductase (KSR) (Figure 1) [16]. Following this process, an acyl-CoA chain will be added to sphinganine to form dihydroceramides, and this process is facilitated by a group of ER resident enzymes known as ceramide synthases (CerS) (Figure 1) [16]. Lastly, dihydroceramides are converted to ceramides by dihydroceramide desaturase 1 (DES1) (Figure 1) [16].

The salvage pathway involves the degradation of glucosylceramide, sphingomyelin, and other complex sphingolipids, in acidic organelles such as the lysosome or late endosome, to form ceramides (Figure 1) [17]. These ceramides are then broken down by acid ceramidase to form a sphingosine base and a free fatty acid chain (Figure 1). The sphingosine base can exit the lysosome, and ceramide can be formed by the attachment of a fatty acid chain to the sphingosine base by CerS, in the ER (Figure 1) [17]. In the sphingomyelinase pathway, ceramides are derived from the hydrolysis of sphingomyelins by neutral sphingomyelinase (nSMase), an enzyme found in Golgi, microsomal, plasma membrane, and nuclear fractions (Figure 1) [17,18].

CerSs, as highlighted previously, are a group of ER-resident enzymes which are important gatekeepers of ceramide levels, as they largely account for ceramides from the de novo and salvage pathways [19,20]. There are a total of six mammalian ceramide synthases (CerS1-6), with each CerS member showing a substrate preference for different lengths of fatty acid chains and the subsequent acyl length of ceramides (Table 1) [19,20,21,22,23,24]. Importantly, the expression of the mammalian CerS differs in various types of tissue. CerS1 is abundant in mouse brain and skeletal tissues; CerS2 is ubiquitously expressed in most mouse tissue types, and is most abundant in mouse liver and kidney tissues; CerS3 has the highest expression in mouse testis and skin tissues; CerS4 is most highly expressed in mouse skin, leukocytes, heart, and liver tissues; CerS5 is expressed at low levels in most tissue types, with slightly higher expression found in skeletal muscle, testis, and kidney tissues; CerS6 is expressed at low levels in most tissues, with slightly higher expression in intestine and kidney tissues [22]. This difference in CerS expression in various tissues may perhaps explain the variation in ceramide species and their relative abundance in different tissue types. In addition, the dysregulation of these CerS expression in specific tissues would implicate the development of disease (Table 1) [25,26,27,28,29,30,31,32,33].

## 3. Ceramides in Disease

Ceramides are linked to numerous diseases, but it is important to appreciate that its reported diverse impact on pathophysiology is related to the length of its fatty acid chain moiety. For example, ceramides are commonly associated with skin diseases as it is suggested to constitute approximately 50% of all lipids in the stratum corneum (SC) of the skin [34]. In atopic dermatitis (AD), the loss of a proper water barrier was found to be associated with altered lipid composition in the SC. Ceramides with ultra-long fatty acyl chains of >24 carbon atoms (C25 and above) were reduced, while the long acyl chain ceramides (C24 and below), were increased in the SC of AD patients [35,36]. This suggests that an increase in skin long chain ceramides at the expense of ultra-long chain ceramides may contribute to skin pathologies [35,36]. Indeed, the dysregulation of ceramide profiles in AD was supported by a randomized control trial (RCT) on 18 female participants with AD, where the topical application of a ceramide cream significantly ameliorated the severity of skin lesions after 4 weeks of treatment [37]. In another trial conducted by Spada and colleagues, transepidermal water loss (TEWL) was shown to be significantly lower in eczema patients on a ceramide-dominant moisturizing cream and cleanser when compared to the placebo group. Despite no significant difference in the assessment of the eczema area severity index (EASI) between the two groups [38], it does highlight the importance of (restoring) ultra-long acyl chain ceramides in regulating water balance in the skin. Such an observation was further corroborated by Ito et al., who showed that ceramide synthase 4 (CerS4), a key enzyme in the biosynthesis of long acyl chain ceramides, C18–C20 ceramides, was significantly higher in changed skin (affected sites) of AD patients compared to unchanged skin sites [29]. Conversely, mice lacking CerS3, an enzyme involved in the biosynthesis of ultra-long acyl chain ceramides (C26–C34 ceramides), exhibited more than a two-fold increase in TEWL rate than in control mice, with a concomitant reduction in C26-, C28-, and C30-ceramides, highlighting that loss of ultra-long acyl chain ceramides accompanied epidermal water permeability barrier disruption [39]. An autosomal recessive mutation leading to the inactivation of CerS3 was also reported to result in congenital ichthyosis, further highlighting the importance of CerS3 and the synthesis of ultra-long ceramides in maintaining skin physiology [28].

Within domains of obesity and type-2 diabetes mellitus (T2DM), an increase in C18- and C16-ceramides are associated with metabolic dysfunction, again linking long acyl chain ceramides to pathology. The knockout of CerS1 in the skeletal muscle of C57BL/6 mice was reported to prevent C18-ceramide accumulation with improvements in insulin resistance and glucose tolerance [25]. Pancreatic beta-cells are particularly sensitive to changes in extracellular lipids. Under glucolipotoxic conditions of T2D, CerS4 expression was upregulated, with parallel increases in C18-, C22-, and C24:1-ceramides in the rat insulinoma cell line INS-1 [30]. Similarly, high glucose and palmitate increased expression of CerS5 and CerS6 in the mouse pancreatic beta cell line MIN6, and knockdown of either CerS5 or CerS6 abrogated the glucolipotoxic response [32]. Separately, a global knockout of mouse CerS5 reduced C16:0 ceramide levels in muscle, liver, and epididymal white adipose tissues, leading to reduced high-fat diet-associated insulin insensitivity and glucose intolerance [31]. CerS6 (the enzyme that synthesizes C14 and C16 ceramides) expression was elevated in adipose tissues of obese humans, while there was global ablation of CerS6 in mice protected against glucose intolerance when challenged with a high-fat diet [33]. Similarly, higher levels of plasma and hepatic C16 ceramides, as well as higher levels of CerS6 expression, were found in liver and subcutaneous adipose tissues of male Ob/Ob mice [40]. Separately, Kim and colleagues showed that C57BL/6 mice on a high-fat diet for 18 weeks had elevated hepatic expression of CerS1, CerS5, and CerS6, and the latter coincided with increased hepatic C16 ceramide levels and liver steatosis [41].

This detrimental impact of long acyl chain ceramides on T2DM was corroborated by several human cohort studies. A 6-year longitudinal population study revealed that several circulating ceramides, including C18:1-ceramide, C20-ceramide, C20:1-ceramide, and C22:1-ceramide were found to be positively associated with T2DM incidence and inversely associated with HOMA-B, the latter an assessment of the pancreatic β-cell function [42]. Similarly, a prospective study showed that higher levels of plasma C16-, C18-, C20-, and C22- ceramides were each associated with increased risk of developing T2DM [43]. Independently, a higher C18-ceramide/C16-ceramide ratio in the plasma was found to be a predictor of T2DM, with C18-ceramide levels correlated with T2DM incidence [44]. Wittenbecher et al. also found that plasma C18-ceramide, C22-ceramide, C20-dihydroceramide, and C22-dihydroceramide were associated with T2DM incidence [45].

Indeed, long acyl chain ceramides are linked to cardiovascular diseases. The accumulation of endothelial cell long acyl chain ceramides in high-fat diet-fed mice was linked to vascular dysfunction. The inhibition of ceramide generation from the de novo synthesis pathway, through the heterozygous deletion of DES1, was sufficient to ameliorate the endothelial nitric oxide synthase (eNOS) activity and vascular dysfunction [46]. Inhibition of ceramide synthesis by myriocin, a pharmacological inhibitor of serine palmitoyl transferase (SPT), was also shown to be beneficial in reducing atherosclerotic lesions in high-fat diet-fed apolipoprotein E (apoE) knockout mice [47,48]. Similarly, the inhibition of ceramide synthesis protected against heat failure, as the myriosin treatment of LpL^GPI^ mice (overexpressing glycosylphosphatidylinositol (GPI)-anchored human lipoprotein lipase specifically in the heart, leading to higher lipid uptake and cardiomyopathy) improved heart function and lowered gene expression of heart failure-associated genes such as *Glut1*, *Glut4*, *Pdk4*, *Cd36*, *Acs1*, and *Fatbp1* [49].

Dysregulation of plasma ceramide levels were also reported to be correlated to the development of cardiovascular diseases [45,50]. A prospective study performed on a European cohort found that plasma C16-ceramide, C18-ceramide and C24:1-ceramide were associated with increased risk of coronary artery disease [50]. Similarly, a separate cohort study also reported that C16-ceramide and C22:2 dihydroceramide were associated with higher risk of cardiovascular diseases [45]. In another study, C16:1-, C16:0-, and C24:1- ceramides were found to be elevated in myocardium samples obtained from advanced heart failure patients when compared to control myocardial samples [27]. Elevated *CerS1* expression (involved in synthesis of C18-ceramide) was also found in cardiac tissues of patients with heart failure when compared to tissues from non-diseased patients [26]. In a study looking at the effects of liraglutide treatment, a glucagon-like peptide-1 agonist known to reduce CVD risk in T2DM patients, plasma ceramides with acyl lengths of 19 and 20 carbon atoms were lowered by GLP-1 treatment, suggesting that the lowering of long acyl chain ceramides may contribute to reported GLP-1 cardio-protection [51]. Indeed, a study performed on European and American cohorts found that a higher ratio of ultra-long acyl chain ceramides, C24-ceramide, to long acyl chain ceramides, C16-ceramide, was inversely correlated with coronary heart disease and heart failure [52].

## 4. Linking Circulating Ceramides with Intracellular Lipid Regulation

It has become increasingly clear that the levels of circulating ceramides are intricately linked to intracellular lipid regulation, particularly at the ER, due to the organelle localization of the various ceramide synthases. Apart from this de novo synthesis pathway, ceramides can be taken up by cells with shorter chain ceramide species, such as C2- and C6-ceramides being far more efficient at passing through the plasma membrane compared to ceramides with an acyl chain length of 16 carbons and above [53,54]. Hence, when studying the contribution of ceramides to cell physiology and pathology, it is important to consider them against a backdrop of intracellular lipid regulation and the activity of ER fatty acid enzymes. Circulating lipids remain the reservoir of substrates for ceramide synthesis [55]. However, lipids, especially free fatty acids (FFAs), have a preferred evolved route of shuttling into the ER for either assembly/esterification into neutral lipids or for generation of metabolic intermediates and signalling molecules [56,57]. The availability and retention of lipids within the ER is therefore very much dependent on the activity of enzymes and scaffold proteins that are responsible for shuttling fatty acids and other lipid intermediates into other compartments such as the Golgi apparatus, lipid droplets, peroxisomes, and/or lysosomes.

## 5. Are Ceramides Responsible for Certain Fatty Acid-Induced ER Stress and Apoptosis?

Certain ceramides, produced after exposure to exogenous fatty acids such as palmitate, were found to induce pancreatic β-cell ER stress and apoptosis. Zheng et al. showed that C16 ceramides accompany heightened ER stress and apoptosis when lipid droplet biogenesis was disabled [58]. Fumonisin B1, a non-specific inhibitor of CerS, reduced ER stress in palmitate-exposed MIN6 cells [58]. Manukyan et al. showed that inhibition of C14- and C16-ceramide formation, using either fumonisin B1 or CerS5/CerS6-knockdown, rescued palmitate-induced apoptosis in mouse insulinoma cells and human pancreatic islets [32]. Similarly, the treatment of a rat myoblast cell line, L6 cells, with palmitate was also found to increase C18-ceramides, with a concomitant induction of caspase-3 activity [59]. The inhibition of ceramide biosynthesis in L6 cells, with fumonisin B1, protected against palmitate-induced apoptosis, and cells co-treated with fumonisin B1 and palmitate had significantly lower caspase-3 activity when compared to cells treated with palmitate alone [59]. Similarly, palmitate treatment of mouse C2C12 myoblast cells elevated C16-ceramides with a corresponding induction of apoptosis [60]. Importantly, this impact of ceramide accumulation was reported to be associated with insulin resistance, as the prevention of C18-ceramide accumulation in the muscle, through either global or skeletal-specific knockout of CerS1, significantly improved glucose homeostasis and insulin sensitivity in high-fat diet-fed mice [25].

C16-ceramide accumulation was also linked to hepatocyte ER stress following palmitate treatment [41]. Either overexpression of CerS6 or the exogenous addition of C16- and C18-ceramides exacerbated palmitate-induced ER stress in liver cell lines with an induction of UPR markers, including p-eIF2a, p-PERK, CHOP, and GRP78 proteins [41]. Interestingly, the overexpression of CerS2, which catalyses the formation of ultra-long acyl chain ceramides (C24- and C26-ceramides), protected the cells against palmitate-mediated ER stress [41]. In a separate study, the inhibition of the ceramide de novo biosynthesis pathway, through the intraperitoneal injection of myriosin, ameliorated hepatic apoptosis and liver inflammation in high-fat diet-fed rats, with reduced cleaved caspase-3, TNFα, IL-1β, and IL6 in liver tissues of myriocin-treated steatosis rats [61].

Similarly, C16-ceramides drive apoptosis in human coronary artery endothelial cells (HCAECs). Under hyperglycemic conditions, Zietzer and colleagues showed that C16-ceramides dominated HCAEC-derived large extracellular vesicles (lEVs), the latter of which triggered HCAEC apoptosis [62]. Inhibition of neutral sphingomyelinase 2 (nSMase2), significantly reduced C16-ceramides (as well as other less dominant ceramide species) in lEVs, which resulted in the abrogation of hyperglycemic-induced apoptosis in HCAECs [62].

## 6. Mechanisms Underpinning Ceramide-Mediated Apoptosis

Ceramides were shown to activate both extrinsic and intrinsic apoptotic pathways in various cancer models [63,64,65,66,67,68,69,70,71]. In the extrinsic apoptotic pathway, the generation of ceramides was linked to the activation of Fas-, as well as tumor necrosis factor-related apoptosis-inducing ligand (TRAIL)-dependent apoptotic cascades in cancer cells [63,64,72]. Furthermore, ceramides were observed to reduce abundance of FLICE inhibitory proteins (FLIPs), an inhibitor of caspase 8, in glioblastoma and renal carcinoma cells [65,66].

In the intrinsic apoptotic pathway, ceramides were suggested to form large channels on the outer membrane of the isolated rat liver mitochondria, leading to increased permeability of small proteins, including cytochrome c, and triggering of the apoptotic cascade [73]. Furthermore, ceramides were shown to induce the phosphorylation of p38 mitogen-activated protein kinase (MAPK), leading to de-phosphorylation (inactivation) of Akt, and subsequent translocation of pro-apoptotic protein, Bax, to the mitochondria. Bax then facilitates cytochrome c release from the mitochondria into the cytosol, triggering apoptosis [68]. In addition, ceramides were observed to downregulate the Akt signaling cascade by binding to the inhibitor of protein phosphatase 2A (I2PP2A), leading to the activation of protein phosphatase 2A (PP2A), and subsequent inhibition of Akt [67,74]. Activated PP2A is linked to the de-phosphorylation (inactivation) of the anti-apoptotic Bcl2 protein [69]. Ceramides were separately linked to reduced levels of the anti-apoptotic protein, survivin, leading to increased expression of pro-apoptotic *Bax* [70,71].

As mentioned earlier, the acyl length of ceramides is linked to its toxicity, with C16- and C18-ceramides being notorious in the development of various diseases [29,32,41,62]. Saddoughi et al. reported that I2PP2A preferentially binds to C18-ceramides over C14-, C16-, C20-, C22-, and C24-ceramides [75], suggesting that C18-ceramides are a relatively more potent inhibitor of Akt compared to other ceramides. Hence, C18 ceramides may activate downstream apoptotic pathways more effectively. It was also reported that only C16-ceramides are able bind to p53, preventing its degradation by the E3 ubiquitin ligase, MDM2 [76]. Furthermore, the transient overexpression of CerS6 resulted in the stabilisation of p53 protein, while CerS1-5 overexpression did not [76]. This stabilisation of p53 could potentially increase binding of p53 to Bcl2, facilitate Bax translocation to mitochondria, and lead to the subsequent activation of apoptosis [69]. Separately, the formation of a mitochondrial outer membrane pore by C16-ceramides was found to be disrupted by ultra-long acyl chains (C22- and C24-ceramides) [77]. Conversely, such ultra-long acyl chain ceramides could also induce the formation of smaller membrane channels which were vice versa disrupted by increased C16-ceramides [77]. Therefore, apart from its interaction with proteins linked to apoptosis, the channel forming function of long acyl chain- and ultra-long acyl chain-ceramides perhaps require further investigation, especially with regards to the disruption of mitochondrial permeability and cytochrome c release.

## 7. Lipid Droplet Biogenesis: A Potential Protective Mechanism against C18- and C16-Ceramide Accumulation?

While inhibitors of ceramide biosynthesis, such as the mycotoxin fumonisin B1, was demonstrated to be effective in preventing ceramide-mediated apoptosis in vitro, there were concerns of adverse effects in vivo, especially inflammation and necrosis of the liver and kidney in mice [78]. Apart from the direct modulation of ceramide synthase, handling of excess intracellular fatty acids may offer another avenue for preventing long-chain ceramide accumulation, ER stress, and apoptosis in cells. Proper sequestration of fatty acids as neutral lipids, such as triglycerides, within lipid droplets (LDs) could prevent ceramide accumulation as it reduces the availability of free fatty acid substrates at the ER for ceramide synthesis. This was evident from a study performed by Zheng and colleagues, where the reduction of LD biogenesis, through the β-cell specific knockout of FITM2 protein in high-fat diet-fed mice, resulted in a significant increase in C16-ceramides in β-cells [58]. Similarly, the disruption of LD biogenesis, through the adipose-specific knockout of SEIPIN, also resulted in a significant increase in C18- and C20-ceramides [79].

Indeed, the sequestration of palmitate, the most abundant saturated fatty acid found in human serum, in the form of neutral lipids was found to protect the cells against palmitate-mediated apoptosis in multiple studies [80,81,82,83,84,85,86]. Exposure of the unsaturated fatty acid, oleate, prevented palmitate-induced apoptosis by promoting the incorporation of palmitate into triglycerides in CHO-, 1.1B4-, and INS1e cells, with increased intracellular neutral lipid accumulation [81,82,83]. In addition to oleate, the polyunsaturated fatty acid, C20:4 arachidonic acid, was also reported to have a similar beneficial impact on C2C12 cells, a mouse skeletal muscle cell line [84]. The exogenous addition of linoleate, oleate, α-linolenate, and docosahexaenoate to microglial cells also protected the cells from palmitate-induced cell death, with a concomitant increase in neutral lipid formation [85]. Apart from its effect on the sequestration of FFAs, LDs were also reported to store ceramides, in the form of acylceramide, in the livers of high-fat diet-fed mice [86].

LDs facilitate sequestration of ceramides, and therefore the stabilization of protein targets involved in LD formation may mitigate ceramide-induced apoptosis. The formation of LD occurs at the ER and begins with the accumulation of triglycerides between the ER membranes, and the subsequent curving and formation of an oil lens. With sufficient accumulation of triglycerides, the oil lens will then bud off from the ER as LD [87]. LD biogenesis involves many proteins, but three ER proteins—seipin, perilipin, and FITM2—are critical for LD formation. Seipin recruits and promotes the accumulation of triglycerides at the LD biogenesis site, while perilipins (PLIN2 and PLIN3) protect these triglyceride aggregates from lipolysis [88,89]. FITM2, other than its acyl coA diphosphatase activity, promotes membrane curvature at the LD biogenesis sites by interacting with ER tubule-forming proteins and septins [90,91].

## 8. Conclusions

Emerging data point towards the benefit of accelerating lipid droplet biogenesis to sequester fatty acid substrates away from CerS1 and CerS4–CerS6 in the ER. This could lower the generation of long acyl chain length ceramides, notably C18- and C16-ceramides. However, this is predicated upon rapid hydrolysis of the lipid droplets by the mitochondria to prevent lipid droplet accumulation, which may result in steatosis. Hence, much more research is needed to understand the evolutionary importance of long acyl chain ceramides and on how knowledge on intracellular lipid regulation can be harnessed to dampen its negative impact on various cells.

## Figures and Tables

**Table 1:** The different ceramide synthases (CerS) characterized to date, their substrate preferences, and their associations with different diseases.

CerS	Acyl Chain Length Preference	Disease Implicated	Reference
CerS1	C18	↑ Type 2 Diabetes: Skeletal muscle	Turpin-Nolan et al., 2019 [25]
		↑ Heart Failure: Myocardium	Carrillo et al., 2021 [26]
CerS2	C22–C24	↓ Heart Failure: Myocardium	Ji et al., 2017 [27]
CerS3	C26–C34	↓ Congenital Ichthyosis: Skin	Eckl et al., 2013 [28]
CerS4	C18–C20	↑Atopic dermatitis: Skin	Ito et al., 2017 [29]
		↑ Type 2 Diabetes: β-cells	Véret et al., 2011 [30]
CerS5	C14–C16	↑ Obesity and Type 2 Diabetes: White adipose tissue	Gosejacob et al., 2016 [31]
		↑ Type 2 Diabetes: β-cells	Manukyan et al., 2015 [32]
CerS6	C14–C16	↑ Obesity and Type 2 Diabetes: White adipose tissue, Liver	Turpin et al., 2014 [33]
		↑ Type 2 Diabetes: β-cells	Manukyan et al., 2015 [32]

## Data Availability

Not applicable.
